# T Cell Receptor (TCR)-Induced PLC-γ1 Sumoylation via PIASxβ and PIAS3 SUMO E3 Ligases Regulates the Microcluster Assembly and Physiological Function of PLC-γ1

**DOI:** 10.3389/fimmu.2019.00314

**Published:** 2019-02-28

**Authors:** Qi-Long Wang, Jia-Qi Liang, Bei-Ni Gong, Ji-Ji Xie, Yu-Ting Yi, Xin Lan, Yingqiu Li

**Affiliations:** ^1^MOE Key Laboratory of Gene Function and Regulation, State Key Laboratory of Biocontrol, Sun Yat-sen University, Guangzhou, China; ^2^School of Life Sciences, Sun Yat-sen University, Guangzhou, China

**Keywords:** sumoylation, TCR-proximal signaling, PLC-γ1, microcluster, PIAS SUMO E3

## Abstract

The SUMO modification system plays an important role in T cell activation, yet how sumoylation regulates TCR-proximal signaling remains largely unknown. We show here that Phospholipase C-γ1 (PLC-γ1) is conjugated by SUMO1 at K54 and K987 upon TCR stimulation and that K54 sumoylation is pivotal for PLC-γ1-mediated T cell activation. We further demonstrate that TCR-induced K54 sumoylation of PLC-γ1 significantly promotes the formation of PLC-γ1 microclusters and the association of PLC-γ1 with the adaptor proteins SLP76 and Gads, but only slightly affects the phosphorylation of PLC-γ1 on Y783, which determines the enzyme catalytic activity. Moreover, upon TCR stimulation, the SUMO E3 ligases PIASxβ and PIAS3 both interact with PLC-γ1 and cooperate to sumoylate PLC-γ1, facilitating the assembly of PLC-γ1 microclusters. Together, our findings reveal a critical role of PLC-γ1 K54 sumoylation in PLC-γ1 microcluster assembly that controls PLC-γ1-mediated T cell activation, suggesting that sumoylation may have an important role in the microcluster assembly of TCR-proximal signaling proteins.

## Introduction

TCR signaling controls the adaptive immune response. TCR signaling is initiated by ligation of the TCR and its coreceptors (e.g., CD28, CD4, and CD8) by cognate antigens bound to MHC molecules (pMHC) presented by antigen-presenting cells. Briefly, TCR ligation triggers phosphorylation of ITAMs (immunoreceptor tyrosine-based activation motifs) on CD3 and the ζ-chains of the TCR complex by Lck (lymphocyte-specific protein tyrosine kinase) (1, 2). The phosphorylated ITAM tyrosines serve as docking sites to recruit and activate Zap70 (ζ chain of TCR-associated tyrosine kinases of 70 kD), which then phosphorylates the adaptor protein LAT (linker for activation of T cells), a plasma membrane-localized adaptor protein, at multiple tyrosines (3, 4). Tyrosine-phosphorylated LAT associates with multiple proteins, such as PLC-γ1 (phospholipase C-γ1) and Gads (Grb2-related adaptor downstream of Shc). Gads further binds to the adaptor protein SLP76 (SH2 domain-containing leukocyte protein of 76 kDa) (5, 6). Following TCR ligation, tyrosine kinase ITK (interleukin-2-inducible T-cell kinase) is recruited to PIP3-rich membranes in which ITK interacts with the SLP76/LAT complex and is activated by Lck; then, the activated ITK interacts with and activates PLC-γ1, which in turn catalyses PIP2 to produce the second messengers IP3 and DAG that trigger calcium influx and activation of MAPKs and PKC, eventually leading to the activation of a number of transcription factors, notably NFAT, NFκB, and AP-1 (3, 7, 8).

To play crucial roles in implementing rapid and accurate responses to different ligands with a wide range of affinities and in securing appropriate outcomes in T-cell development, homeostasis, activation, acquisition of effector functions and apoptosis, TCR signaling has evolved diverse, multi-layered regulatory mechanisms (3, 5, 9–11). Microscopic images have revealed the conjugation structure called immunological synapse formed between T-APC cell and microclusters (signaling complexes, 200~500 nm) of TCRs, coreceptors, TCR-proximal kinases and adaptor proteins that serve as centers of signaling within immunological synapse (5, 12–14). These microclusters that form at the plasma membrane following TCR stimulation are heterogeneous and dynamic and are assembled by multivalent interactions between protein complexes, facilitating TCR signaling through spatial phase separation to generate a distinct physical and biochemical compartment (5, 9, 13). Further exploration of novel mechanisms in TCR-proximal signaling, especially microcluster formation, and the functional consequences will provide new insight into T cell immunity and supply potential targets for immunotherapy.

SUMO (small ubiquitin-related modifier) modification is a reversible post-translational modification that is a key regulatory mechanism for a large variety of fundamental cellular processes, such as chromatin organization, transcription, DNA repair, macromolecular assembly, protein homeostasis, trafficking, and signal transduction (15). At the molecular level, SUMO modification can mask or enhance the binding site of its target to alter the molecular binding ability of the target protein (16). There are at least four SUMO isoforms in humans, SUMO1-4, each of which is encoded by a distinct gene and is highly conserved in all eukaryotes (17). The proteins SUMO1-3 are expressed as precursors and need to be proteolytically processed to make the C-terminal Gly-Gly motif available for conjugation, yet the maturation and conjugation of SUMO4 are still under debate (18). Mature SUMO2 and SUMO3 are often referred to as SUMO2/3 because they share 97% identity. In contrast, SUMO1 shares only ~50% identity with SUMO2/3 (19). Akin to that of ubiquitin, the conjugation of SUMO proteins to substrates occurs through three enzymatic steps involving the sequential reactions of an E1 activating enzyme, an E2 conjugating enzyme, and an E3 protein ligase. In the final step, SUMO is conjugated to the ε-amino group of a lysine residue of the target protein (19). The cysteine proteases of the SENP (sentrin-specific protease) family rapidly deconjugate SUMO modifications; this deconjugation makes sumoylation very transient and causes only a small fraction of the substrate to be present in a modified form at any one time, making sumoylation difficult to study compared to some other post-translational modifications (20, 21). Sumoylation has been demonstrated to regulate T cell development and T regulatory cell function by targeting STAT5 and the transcription factor IRF4 (22, 23). Our previous work demonstrated that TCR-induced sumoylation of PKC-θ is essential for the formation of mature immunological synapses and for T cell activation (11, 24). To date, PKC-θ is the only identified substrate of sumoylation in TCR-proximal (upstream) signaling.

In this study, we found that PLC-γ1 could be sumoylated at K54 and K987 upon TCR stimulation and that a sumoylation-deficient PLC-γ1 K54R but not K987R mutant significantly impaired TCR-induced Ca^2+^ signaling, NFAT activation and IL-2 production. Furthermore, we found that desumoylation of PLC-γ1 inhibited the microcluster formation of PLC-γ1 and its interaction with the adaptor proteins SLP76 and Gads. By fusing SUMO1 respectively, to the N- and C-terminus of PLC-γ1, we proved that SUMO1 modification in PH domain is important for PLC-γ1 activation. We also identified PIASxβ and PIAS3 as the SUMO E3 ligases for PLC-γ1. In summary, this study characterized PIASxβ/3-mediated sumoylation of PLC-γ1 in TCR-proximal signaling and demonstrated its important role in PLC-γ1 microcluster assembly and T cell activation.

## Materials and Methods

### Plasmids, shRNAs, and siRNAs

PLC-γ1 (NCBI Reference Sequence: NM_002660.2) was amplified from Jurkat E6.1 cells cDNA and cloned into the pFlag-CMV-2 (Sigma) and pcDNA3.1-HA (Invitrogen), respectively. The point mutations K54R and K987R were mutated using pfuUltra II DNA polymerase according to the manufacturer's instructions (Stratagene, San Diego, CA, USA). The PIASxβ and PIAS3 shRNA and siRNA sequences were designed according to the Invitrogen Block-iT RNAi Designer. Scrambled control nucleotides (5′-CGCTAATTCGACTCGGATA-3′), shPIASxβ sequence (5′-GCCCACGAGTTTAGTTCAA-3′), and shPIAS3 sequence (5′-GCACTGATCAAGGAGAAAT-3′) were constructed into RNA interference expression vector pSUPER.Retro.Neo-GFP (OligoEngine, Seattle, WA, USA), respectively. The scrambled control siRNA, including siPIASxβ (GCCCACGAGUUUAGUUCAA), siPIAS3 (5′-GCCAGGAGCCAAAUGUGAU-3′), siPLC-γ1-1 (5′-GGACUUAGUUUGUGAUGUA-3′), and siPLC-γ1-2 (5′-GUACUGUGUUUCGCAUUAA-3′), were synthesized by RiboBio Co. Ltd (Guangzhou, China).

### Antibodies and Regents

Antibodies to Myc (9E10), HA (Y-11), GFP (B-2), PLC-γ1 (B-4), LAT (FL-233), SLP76 (F-7), Gads (G-11), ERK1 (C-16), PIASxβ (S-15), PIAS3 (H-169), actin (C4), and Lck phosphorylated at Try394 (SC-101728) were from Santa Cruz Biotechnology. Antibodies to SUMO1 (ab32058), PIASxβ (ab88598), SLP76 phosphorylated at Try145 (ab75829), LAT phosphorylated at Try132 (ab4476) and Try226 (ab14502) were from abcam. M2 antibody to Flag (F3165) was from Sigma-Aldrich (St Louis, MO, USA). Antibody specific for ERK phosphorylated at Thr202 and Tyr204 (E10) and LAT phosphorylated at Try191 (3584) were from Cell Signaling Technology. Mouse anti-TCR (C305, IgM) was from Millipore. Anti-human CD3 (UCHT1), anti-human CD28 (CD28.2), and PLC-γ1 phosphorylated at Try783 (612464) were from BD Pharmingen. Anti-mouse CD3 (17A2) and anti-mouse CD28 (37.51) were from BioLegend. Goat anti-mouse IgG (31160) was from Thermo Fisher Scientific. Horseradish peroxidase-conjugated secondary antibodies were from Jackson ImmunoResearch. PE conjugated anti-human IL2 (130-091-646) was from Miltenyi Biotec (Bergisch Gladbach, Germany). IL-2 ELISA Ready-SET-Go (88-7025-88) was from eBioscience. Alexa Fluor 488-coupled chicken anti-mouse (A-21200), Alexa Fluor 594-coupled goat anti-mouse (A-11005), Alexa Fluor 647-coupled goat anti-rabbit (A-21247) and Alexa Fluor 647-coupled donkey anti-goat (A-21447) were from Invitrogen.

### Cell Culture, Transfection, and Stimulation

Human leukemia Jurkat T cell line E6.1 cells and TAg cells were cultured in RPMI1640 medium (Hyclone) supplemented with 10% (vol/vol) fetal bovine serum (FBS, Hyclone, Logan, UT, USA), 100 U/ml streptomycin, and 100 U/ml penicillin (Gibco) at 37°C, 5% CO_2_. Cells in a logarithmic growth phase were transfected by nucleofection (Amaxa). In each experiment, cells were transfected with the same total amount of DNA by the addition of the requisite quantity of empty vector. After transfection, cells were incubated in RPMI medium containing 10% FBS without penicillin and streptomycin for 48 h. For stimulation with antibodies, cells were washed with serum-free RPMI1640 medium, and stimulated with 10 μg/ml anti-CD3 and/or 2 μg/ml anti-CD28, which were crosslinked with goat anti-mouse IgG (10 μg/ml). For plate bound stimulations, the 24-well plates were exposed to 2 μg/ml anti-CD3 and 2 μg/ml anti-CD28 in PBS and were incubated overnight at 4°C. The plates were washed three times with PBS and then cells were plated onto the wells for various time points at 37°C. Mice were handled according to guidelines approved by the Animal Care and Ethics committee of Sun Yat-Sen University. Primary T cells were isolated from lymph nodes and spleens of mice using pan T cell isolation kit II (Miltenyi Biotec) and were cultured in RPMI1640 medium supplemented with 10% FBS, 2 mM L-glutamine, 1 mM sodium pyruvate, 100 U/ml streptomycin and 100 U/ml penicillin at 37°C, 5% CO_2_. HEK 293T cells were cultured in DMEM (Hyclone) containing penicillin, streptomycin, and 10% FBS. Transfections were carried out with PEI (Sigma-Aldrich) or Lipofectamine 2000 reagent (Invitrogen).

### Immunoprecipitation and Immunoblot Analysis

Western blotting analysis was performed as previously described. Briefly, cells were lysed in lysis buffer (20 mM Tris-HCl, pH 7.5, 150 mM NaCl, 5 mM EDTA, 5 mM NaPPi, 1 mM Na_3_VO_4_, 1 mM PMSF, 1% NP-40, and 10 μg/ml each aprotinin and leupeptin). In some cases, the buffer was also supplemented with 20 mM N-ethylmaleimide (Sigma) or 0.1% SDS. Whole-cell lysates were incubated overnight with 0.5 μg of the appropriate antibodies, and proteins were immunoprecipitated for an additional 4 h at 4°C with protein G-sepharose beads (GE healthcare) with gentle shaking. The immunoprecipitated proteins were resolved by SDS-PAGE, transferred onto a PVDF membrane and probed overnight at 4°C with primary antibodies, followed by incubation for 1 h at room temperature with horseradish peroxidase (HRP)-conjugated secondary antibodies. Signals were visualized by enhanced chemiluminescence (ECL; GE Healthcare) and were exposed to X-ray film or on the ChemiDoc XRS+ system (Bio-Rad). Densitometry analysis was performed with Image J software.

### Sumoylation Assay

Treated cells were lysed in lysis buffer containing 20 mM N-ethylmaleimide. After clearance by centrifugation, 1% SDS (vol/vol) was added to the supernatants, and proteins were dissociated by being heated for 10 min at 90°C. Samples were diluted (1:10) with lysis buffer, and HA-tagged PLC-γ1 or endogenous PLC-γ1 was immunoprecipitated overnight at 4°C with anti-HA or anti-PLC-γ1, respectively. Immunoprecipitated proteins were extensively washed five times with lysis buffer and were analyzed by immunoblot with anti-SUMO1.

### Luciferase Reporter Assay

Jurkat TAg cells were transiently transfected by nucleofection with a combination of NFAT luciferase reporter plasmid and the indicated plasmids. After 24 h, cells were either unstimulated or stimulated with coated ani-CD3 plus anti-CD28 for 6 h and cell lysates were collected for luciferase reporter assay on Berthold Lumat LB 9507. Luciferase activity of cell lysates was measured with a Promega luciferase assay kit according to the manufacturer's instructions. Cotransfected Renilla luciferase reporter plasmid was used as an internal control. Each experiment was repeated at least three times in all cases.

### Calcium Flux Assay

Jurkat E6.1 cells were counted and the cell concentrations were adjusted to 1 × 10^7^/ml in cell loading medium. Then 5 μl of stock fluo-4 AM dye (Thermo Fisher Scientific) per ml of cells was added and the cells were incubated at 37°C and 5% CO_2_ for 60 min. Gently mix the loading cells every 10 to 15 min to ensure even loading. The fluo4-loaded cells were resuspended at 5 × 10^6^/ml in cell loading medium and rested at 4°C for 20 min, and then incubated at 37°C for 5 min before running. Calcium flux was determined by a FACS assay using Aria II flow cytometer (BD).

### TIRF Microscope

Jurkat E6.1 cells were stimulated on glass slides coated with or without anti-TCR C305. Then, cells were fixed with 4% paraformaldehyde and permeabilized with 0.2% Triton X-100. After blocking, cells were incubated with primary Abs against HA or PLC-γ1 and LAT overnight at 4°C. After several washes in PBS, cells were incubated with Alexa Fluor 488- and Alexa Flour 647-labeled secondary Abs for 1 h at room temperature. The images were obtained using the Leica AM TIRF MC imaging system attached to a Leica DMI 6000 inverted epifluorescence microscope and acquired using the Leica AF software. To assess the fluorescence intensity mean value of PLC-γ1, about 30 cells were randomly selected from each of the three independent experiments and analyzed. To count and evaluate the fluorescent microclusters, Leica AF software was used. The average mean fluorescence intensity in the non-cluster area of the cell was ≤10,000 Gray Value/pixel (1 pixel = 228 nm), the criteria for a microcluster was that the diameter was <2 μm, intensities at the center were ≥22,000 Gray Value/pixel.

### Flow Cytometry

For intracellular IL-2 production analysis, Jurkat TAg cells were transiently transfected by nucleofection with a combination of siPLC-γ1 and the indicated plasmids. After 48 h, Cells were left unstimulated or stimulated with plate-bound anti-CD3 plus anti-CD28 for 8 h. Brefeldin A (Sigma-Aldrich) was also added. Collected cells were permeabilized (Cytofix/ Cytoperm Plus; BD) and stained with PE anti-IL2 at 4°C. IL-2 production was determined by a FACS assay using Calibur flow cytometer (BD).

### Enzyme-Linked Immunosorbent Assay

Jurkat E6.1 cells were stimulated for 48 h with anti-CD3 plus anti-CD28, and the concentration of IL-2 in culture supernatants was determined by enzyme-linked immunosorbent assay according to the manufacturer's instructions (eBioscience). A 96-well plate (Corning Costar) was coated overnight at 4°C with monoclonal antibody to IL-2. Triplicates of IL-2 standards and supernatants from cultured cells were then added to the plate, followed by incubation for 2 h at room temperature. A biotinylated polyclonal antibody to IL-2 was added to the plate, followed by incubation for 1 h at room temperature, and then Avidin-HRP was added, followed by incubation for 30 min at room temperature. The amount of bound avidin was then assessed with TMB peroxidase that was acidified by 2 N H_2_SO_4_. The absorbance of each well at 450 and 570 nm was then measured with a spectrophotometric plate reader (BioTek).

### Statistical Analysis

Statistical analysis was performed with a two-tailed unpaired Student's *t*-test. *P*-values of <0.05 were considered statistically significant. GraphPad 7.0 software and SigmaPlot 12 software were used for graphs and statistical analysis.

## Results

### TCR Stimulation Induces SUMO1 Modification of PLC-γ1 on K54 and K987

**Figure 1:**
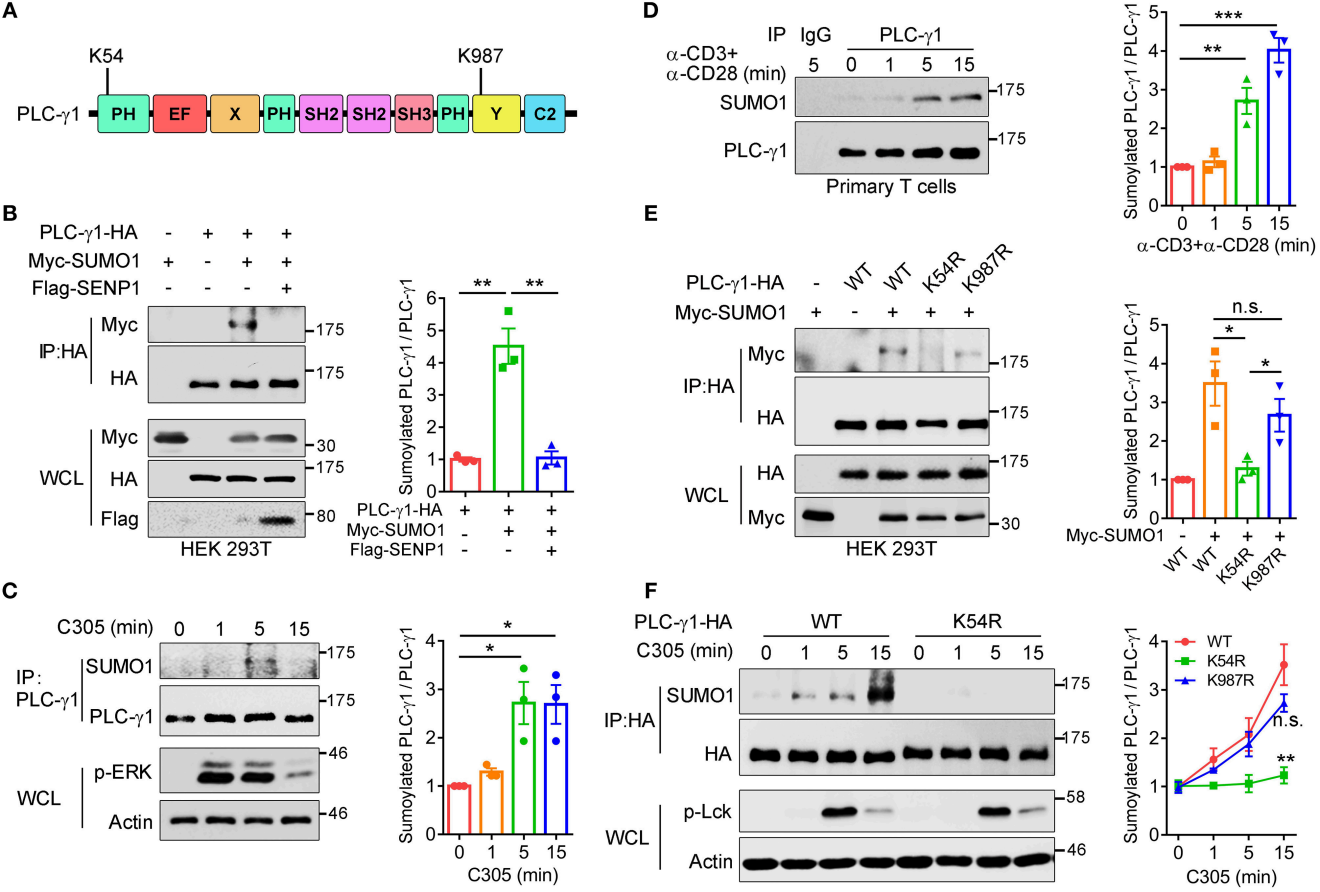
TCR stimulation induces sumoylation of PLC-γ1 on lysine 54 and lysine 987. **(A)** Schematic representation of PLC-γ1. Two predicted sumoylation sites (K54 and K987) are shown. **(B)** Immunoblot analysis of the sumoylation of PLC-γ1 among proteins immunoprecipitated (IP) with anti-HA (top) and immunoblot analysis of whole-cell lysates (WCL) without immunoprecipitation (bottom) with antibodies against various molecules (left margin) in HEK 293T cells transfected with HA-tagged PLC-γ1, Myc-tagged SUMO1 or both together with Flag-tagged SENP1. The densitometric quantification of the ratio of sumoylated PLC-γ1 to immunoprecipitated PLC-γ1 (the ratio for the control was set as 1) is shown on the right. **(C)** Immunoblot analysis of the sumoylation of PLC-γ1 in Jurkat E6.1 cells stimulated with C305 (top) for 0–15 min (above lanes); actin was used as a loading control throughout. The densitometric quantification of the ratio of sumoylated PLC-γ1 to immunoprecipitated PLC-γ1 (the ratio at 0 min was set as 1) is shown on the right. **(D)** Immunoblot analysis of the sumoylation of PLC-γ1 in mouse primary T cells stimulated with plate-bound anti-CD3 and anti-CD28 for 0–15 min. The densitometric quantification of the ratio of sumoylated PLC-γ1 to immunoprecipitated PLC-γ1 (the ratio at 0 min was set as 1) is shown on the right. **(E)** Immunoblot analysis of the sumoylation of PLC-γ1 in HEK 293T cells transfected with HA-tagged PLC-γ1-WT or KR mutants together with Myc-tagged SUMO1. The densitometric quantification of the ratio of sumoylated PLC-γ1 to immunoprecipitated PLC-γ1-WT or KR mutants (the ratio of PLC-γ1-WT was set as 1) is shown on the right. **(F)** Immunoblot analysis of the sumoylation of PLC-γ1 in Jurkat TAg cells transfected with HA-tagged PLC-γ1-WT or PLC-γ1-K54R and then stimulated for 0–15 min with C305. The densitometric quantification of the ratio of sumoylated PLC-γ1 to immunoprecipitated PLC-γ1-WT or KR mutants (the ratio of PLC-γ1-WT at 0 min was set as 1) is shown on the right. n.s.: not significant; ^*^P < 0.05, ^**^P < 0.01, and ^***^P < 0.001 (two-tailed unpaired Student's *t*-test). The data are presented as the mean (± s.e.m.). The data are representative of at least three independent experiments **(B–F)**.

To further understand the importance of SUMO system regulation of TCR-proximal signaling transduction, TCR-proximal signaling proteins were screened for potential sumoylation substrates by using bioinformatic tools. Among them, PLC-γ1 was predicted to contain two ΨKXD/E consensus sumoylation sequences (where “ψ” indicates a hydrophobic amino acid and “D/E” indicates either aspartic acid or glutamic acid) (25), VK^54^LE and TK^987^AE, located at the complete PH domain and Y domain of PLC-γ1, respectively (Figure 1A). Both K54- and K987-containing ΨKXD/E motifs are highly evolutionarily conserved (Supplementary Figure 1A). To investigate whether PLC-γ1 could be sumoylated, HA-PLC-γ1 was coexpressed with Myc-SUMO1 (6 tandem Myc-tagged SUMO1, calculated MW 21.7 kDa, SDS-PAGE MW ~32 kDa) in HEK 293T cells, and a slower-migrating SUMO1-conjugated PLC-γ1 band could be detected and eliminated by coexpression of the main SUMO1 protease SENP1 (26) (Figure 1B). Coexpression of Flag-PLC-γ1 with HA-SUMO1 in HEK 293T cells also led to sumoylation of PLC-γ1 (Supplementary Figure 1B). Further experiments showed that PLC-γ1 was preferentially conjugated by SUMO1 rather than SUMO3 (Supplementary Figure 1C). We next examined whether TCR signaling could induce SUMO1 modification of endogenous PLC-γ1 in T cells. Coimmunoprecipitation analysis in Jurkat T cells showed that endogenous PLC-γ1 was conjugated by SUMO1 following TCR/CD28 costimulation (Supplementary Figure 1D). To exclude non-specific sumoylated protein immunoprecipitated by the binding of protein G beads with anti-CD3 and anti-CD28 IgG antibodies, the anti-TCR IgM antibody C305 was used to stimulate Jurkat T cells, and PLC-γ1 was again conjugated by SUMO1 following TCR (C305) stimulation (Figure 1C). Furthermore, TCR-induced sumoylation of PLC-γ1 was confirmed in murine primary T cells (Figure 1D). Of note, the sumoylated PLC-γ1 was not detected by antibody to PLC-γ1 (Figures 1B–D), indicating a very small fraction of PLC-γ1 could be sumoylated, probably because sumoylated protein states are transient (20, 21). Taken together, these results indicate that PLC-γ1 is sumoylated following TCR stimulation.

To investigate which site contributes to PLC-γ1 sumoylation, we replaced K54 and K987 individually with arginine and assessed the sumoylation of the corresponding KR mutants. Relative to the sumoylation of PLC-γ1-WT (wild type), the sumoylation of PLC-γ1 was completely abolished by K54R mutation but moderately decreased by K987R mutation (Figure 1E), suggesting that sumoylation at K987 may depend on sumoylation at K54 and that K54 is the key SUMO1 conjugation site on PLC-γ1. Next, we determined the contributions of K54 and K987 to TCR-induced sumoylation of PLC-γ1 in T cells. Relative to PLC-γ1-WT, the PLC-γ1-K54R mutant was not sumoylated, whereas PLC-γ1-K987R could be sumoylated to a lesser extent following TCR stimulation (Figure 1F and Supplementary Figure 1E). Together, these results indicate that K54 is the pivotal conjugation site for TCR-induced SUMO1 conjugation on PLC-γ1.

### PLC-γ1 Sumoylation Is Required for T Cell Activation

**Figure 2:**
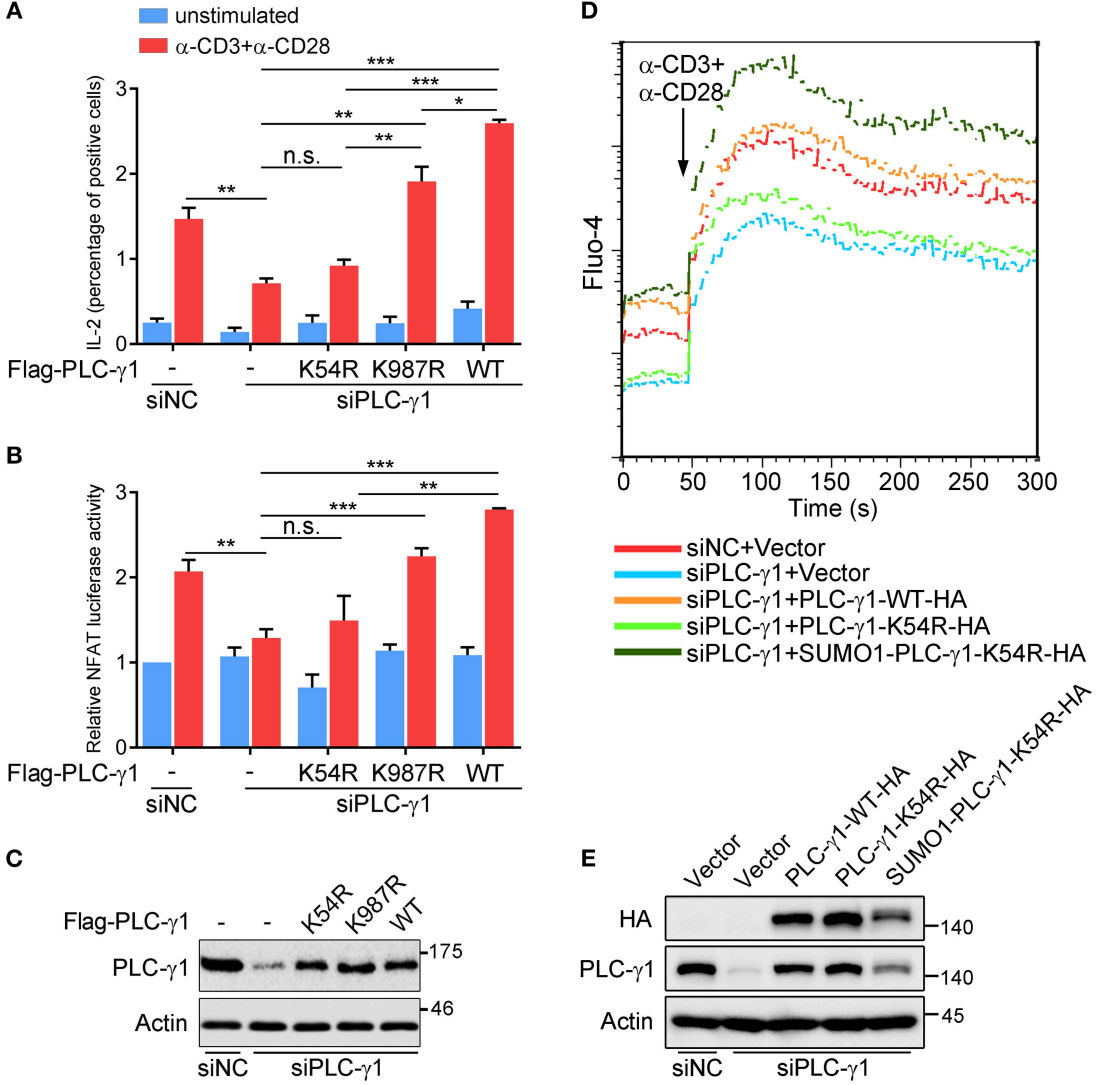
PLC-γ1 sumoylation is required for T cell activation. **(A)** Flow cytometry analysis of the expression of intracellular IL-2 in Jurkat TAg cells transfected with Flag-tagged PLC-γ1-WT or KR mutants together with siPLC-γ1 (siNC served as a negative control) to knock down endogenous PLC-γ1 and then left unstimulated or stimulated with anti-CD3 and anti-CD28 for 8 h. siNC vs. siPLC-γ1, *P* = 0.0062. **(B)** Luciferase reporter assays of Jurkat TAg cells transfected with siPLC-γ1 and Flag-tagged PLC-γ1-WT or KR mutants together with NFAT luciferase reporter plasmids and then left unstimulated or stimulated with anti-CD3 and anti-CD28 for 6 h (the NFAT luciferase activity for negative control left unstimulated was set as 1). PLC-γ1-WT vs. siPLC-γ1, *P* < 0.001; PLC-γ1-K987R vs. siPLC-γ1, *P* < 0.001; PLC-γ1-K54R vs. siPLC-γ1, *P* = 0.3112. **(C)** Expression of PLC-γ1 in Jurkat TAg cells transfected with siPLC-γ1 (siNC served as a negative control) and Flag-tagged PLC-γ1-WT or KR mutants. **(D)** Flow cytometry analysis of the Ca^2+^ flux (fluorescence intensity of Fluo-4) in Jurkat E6.1 cells transfected with siPLC-γ1 (siNC served as a negative control) and HA-tagged PLC-γ1-WT or KR mutants and then stimulated with anti-CD3 and anti-CD28. **(E)** Expression of PLC-γ1 in Jurkat E6.1 cells transfected with siPLC-γ1 (siNC served as a negative control) and HA-tagged PLC-γ1-WT or KR mutants. n.s.: not significant; ^*^P < 0.05, ^**^P < 0.01, and ^***^P < 0.001 (two-tailed unpaired Student's *t*-test). The data are presented as the mean (± s.e.m.). The data are representative of at least three independent experiments.

To further understand the biological relevance of sumoylation on PLC-γ1, we first examined the effect of PLC-γ1-KR mutants on T cell activation by evaluating TCR-induced IL-2 production. Knockdown of endogenous PLC-γ1 by PLC-γ1 mRNA-specific siRNA (siPLC-γ1) significantly inhibited TCR-induced IL-2 production, but reconstituted PLC-γ1-WT and the K987R mutant could completely reverse the defect of IL-2 production, as expected. However, the PLC-γ1-K54R mutant could not restore TCR-induced IL-2 production in siPLC-γ1-treated T cells (Figure 2A and Supplementary Figure 2A). Given that PLC-γ1 mediates TCR-induced activation of NFAT, a key transcription factor of IL-2, we used reporter-gene assays to analyse the importance of PLC-γ1 sumoylation in NFAT activation. In Jurkat T cells transfected with siPLC-γ1 (with siNC as a control), reconstituted PLC-γ1-WT and the K987R mutant, could significantly restore TCR-induced NFAT activation; whereas the PLC-γ1-K54R mutant only slightly but not significantly restored NFAT activation (Figure 2B), although the expression levels of the reconstituted proteins were equal (Figure 2C and Supplementary Figure 2B). We further determined the effect of PLC-γ1 sumoylation on TCR-induced Ca^2+^ flux, which is generated by PLC-γ1. While knockdown of PLC-γ1 in Jurkat T cells with siPLC-γ1 (with siNC as a control) significantly decreased TCR-induced Ca^2+^ flux and transfection with PLC-γ1-WT restored Ca^2+^ flux to normal levels, transfection with PLC-γ1-K54R could only slightly but not significantly reverse the impaired Ca^2+^ flux, which was consistent with its effect in NFAT activation (Figure 2D and Supplementary Figure 2D). Interestingly, reconstituted SUMO1-PLC-γ1-K54R, a mutant that simulates total sumoylation of PLC-γ1 through fusion of SUMO1 to the N-terminus of PLC-γ1-K54R (Supplementary Figure 2C), not only restored Ca^2+^ flux but even caused higher Ca^2+^ flux than reconstituted PLC-γ1-WT did (Figure 2D and Supplementary Figure 2D), although the expression level of SUMO1-PLC-γ1-K54R was much lower than that of PLC-γ1-WT and PLC-γ1-K54R (Figure 2E). Together, these results indicate that SUMO conjugation of PLC-γ1 is essential for its function in T cell activation.

### Sumoylation of PLC-γ1 Promotes Its Microcluster Formation and the Interaction With SLP76 and Gads

**Figure 3:**
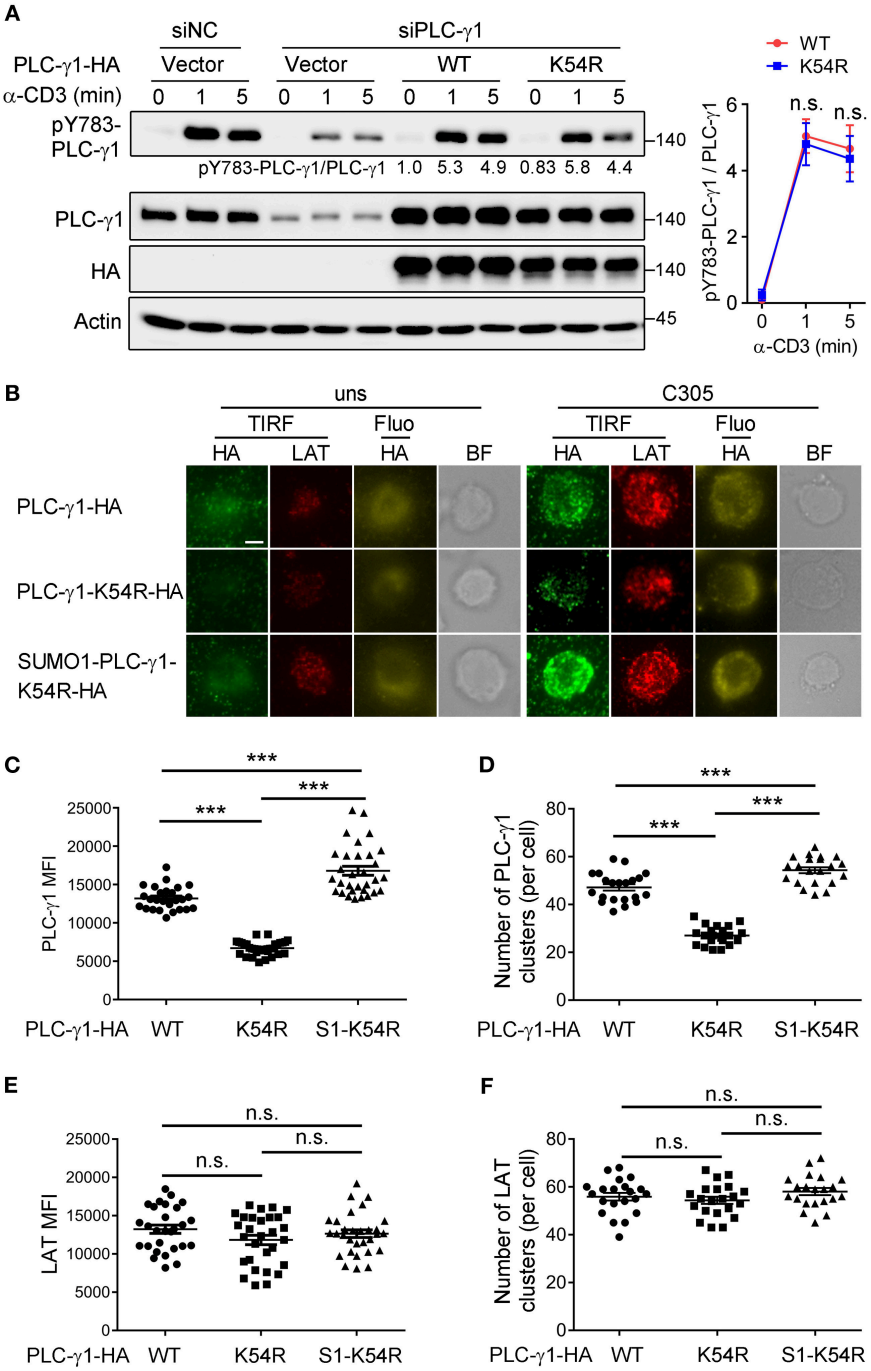
Sumoylation of PLC-γ1 promotes its microcluster formation. **(A)** Immunoblot analysis of the phosphorylation of PLC-γ1 in Jurkat TAg cells transfected with siPLC-γ1 (siNC served as a negative control) and HA-tagged PLC-γ1-WT or PLC-γ1-K54R and then stimulated with anti-CD3 for 0–5 min. The levels of pY783-PLC-γ1 normalized to those of PLC-γ1 are shown. The densitometric quantification of the ratio of pY783-PLC-γ1 to PLC-γ1 (the ratio of PLC-γ1-WT at 0 min was set as 1) is shown on the right. **(B)** TIRF microscopy of the proximal membrane localization of PLC-γ1 (TIRF: green, fluorescence: yellow) and LAT (red) in Jurkat E6.1 cells transfected with siPLC-γ1 and HA-tagged PLC-γ1-WT or KR mutants for 48 h and then left unstimulated (uns) or stimulated with C305 coated on slides for 5 min. Representative images are shown. Scale bar, 4 μm. **(C**–**F)** Quantification of the TIRF mean fluorescence intensity (MFI) of PLC-γ1 **(C)** and LAT **(E)**, and of the number of PLC-γ1 **(D)**, and LAT **(F)** microclusters in cells treated with C305. Each symbol represents an individual cell. S1-K54R: SUMO1-PLC-γ1-K54R. n.s.: not significant; ^***^P < 0.001 (two-tailed unpaired Student's *t*-test). The data are presented as the mean (± s.e.m.). The data are representative of at least three independent experiments.

TCR-induced phosphorylation of PLC-γ1 Y783 is critical for enzyme activation and thus calcium flux (27, 28). To investigate how sumoylation affects PLC-γ1 function, we examined TCR-induced Y783 phosphorylation on PLC-γ1. Interestingly, the K54R mutation of PLC-γ1 did not clearly inhibit TCR-induced Y783 phosphorylation (Figure 3A). Upon TCR engagement, TCR-proximal signaling molecules translocate to the plasma membrane and form activation microclusters. These microclusters contain molecular complexes composed of TCR, signaling enzymes such as ZAP70 and PLC-γ1, and several adaptor proteins, including LAT, SLP76, Grb2, and Gads (29–32). We transfected Jurkat T cells with HA-PLC-γ1-WT or mutants and stimulated cells on glass coverslips coated with or without C305 antibody. Then, we examined the cellular localization of PLC-γ1 by total internal reflection fluorescence (TIRF) microscopy, using endogenous LAT as a control. We found that both before and after TCR stimulation, the ability of PLC-γ1-K54R to assemble into microclusters was much lower than that of PLC-γ1-WT, while SUMO1-PLC-γ1-K54R generated microclusters very well, even better than PLC-γ1-WT (Figures 3B–D). Similar results were obtained when Jurkat T cells were transfected with GFP-tagged PLC-γ1-WT and the K54R mutant (Supplementary Figures 3A–C). Notably, LAT microcluster formation was not affected (Figures 3E,F). Analysis of plasma membranes isolated from T cells through cell fractionation experiments also showed that sumoylated PLC-γ1 was primarily localized in the membrane (data not shown). These observations indicated that sumoylation of PLC-γ1 promoted its localization at the plasma membrane and the formation of microclusters.

To investigate whether the position of sumoylation is important, we constructed a chimeric plasmid with SUMO1 fused to the C-terminus of PLC-γ1-K54R (Supplementary Figure 3D) and examined TCR-induced PLC-γ1 microcluster formation and Ca^2+^ flux in PLC-γ1-depleted cells. Consistently, reconstitution with PLC-γ1-WT or SUMO1-PLC-γ1-K54R resulted in higher PLC-γ1 microcluster formation and Ca^2+^ flux than reconstitution with PLC-γ1-K54R. However, TCR-induced PLC-γ1 microcluster formation and Ca^2+^ flux in Jurkat T cells reconstituted with PLC-γ1-K54R-SUMO1 were similar to those in cells reconstituted with PLC-γ1-K54R (Supplementary Figures 3E–H). These results indicated that sumoylation at the N-terminus of PLC-γ1 is required for its function.

**Figure 4:**
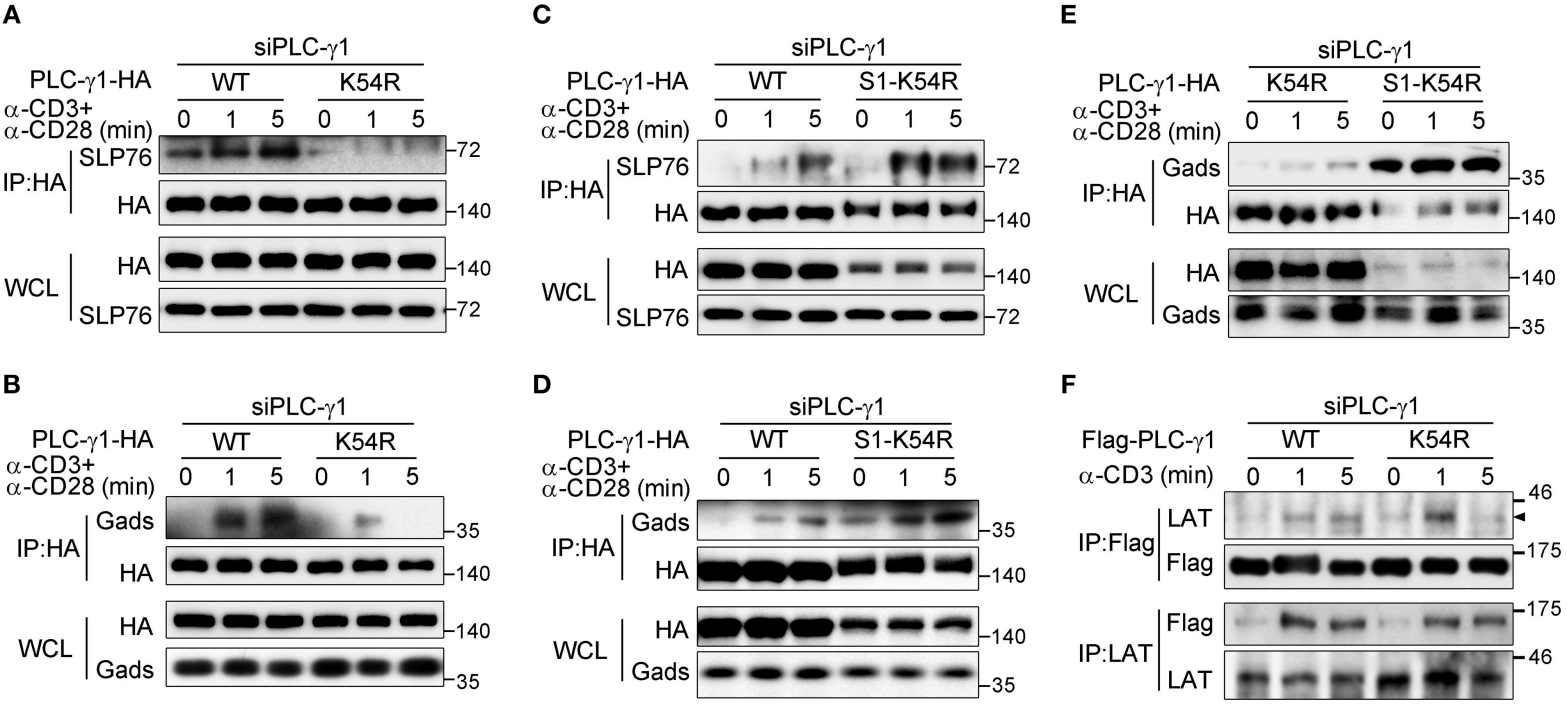
Sumoylation of PLC-γ1 promotes its interaction with SLP76 and Gads. **(A**–**E)** Immunoblot analysis of the association between PLC-γ1 and SLP76 **(A,C)** or Gads **(B,D,E)** among proteins immunoprecipitated with anti-HA from Jurkat E6.1 cells transfected with siPLC-γ1 together with HA-tagged PLC-γ1-WT, PLC-γ1-K54R, or SUMO1-PLC-γ1-K54R (S1-K54R), and stimulated for 0–5 min with anti-CD3 and anti-CD28. **(F)** Immunoblot analysis of the association between PLC-γ1 and LAT among proteins immunoprecipitated with anti-Flag or anti-LAT from Jurkat TAg cells transfected with siPLC-γ1 together with Flag-tagged PLC-γ1-WT or PLC-γ1-K54R and stimulated for 0–5 min with anti-CD3. The data are representative of at least three independent experiments.

Sumoylation can modify the surface of its target, thus changing the molecular binding ability of the target protein (16). LAT is crucial for PLC-γ1 membrane recruitment, and Gads and SLP76 are required for activation of PLC-γ1 via coupling it to ITK (5, 7, 28, 33). We investigated the effect of the K54R mutation on the interaction of PLC-γ1 with these proteins in Jurkat T cells following TCR stimulation. Relative to that of PLC-γ1-WT, the TCR-induced association of PLC-γ1-K54R with SLP76 or with Gads was significantly decreased in Jurkat T cells with knockdown of endogenous PLC-γ1 (Figures 4A,B and Supplementary Figures 4A,B). Interestingly, SUMO1-PLC-γ1-K54R showed a much stronger association with SLP76 or Gads than PLC-γ1-WT or PLC-γ1-K54R did following stimulation (Figures 4C–E and Supplementary Figures 4A,B). However, the K54R mutation did not affect the association between PLC-γ1 and LAT (Figure 4F and Supplementary Figure 4C). We further examined whether the phosphorylation of LAT and SLP76 was affected by their associations with desumoylated PLC-γ1 and found that TCR-induced phosphorylation of LAT on Y132, Y191, Y226, and phosphorylation of SLP76 on Y145 were not significantly affected by desumoylated PLC-γ1 (Supplementary Figures 4D,E), suggesting that PLC-γ1 desumoylation do not affect the upstream phosphorylation of LAT and SLP76. However, in a previous study analyzing the PLC-γ1-deficient cell line Jgamma1 and its reconstituted counterpart, Jgamma1.WT, PLC-γ1 was reported to exert positive feedback on TCR/Zap-70 phosphorylation and negative feedback on SLP76-associated protein phosphorylation (34). The reason we did not observe the feedback effects of PLC-γ1-K54R should be that PLC-γ1 was only desumoylated but not deficient and still associated with LAT.

Together, these results indicate that sumoylation of PLC-γ1 at K54 is required for TCR-induced PLC-γ1 microcluster formation, Ca^2+^ flux, and PLC-γ1 association with SLP76 and Gads.

### PIASxβ and PIAS3 Are the SUMO E3 Ligases of PLC-γ1 and Regulate PLC-γ1 Microcluster Formation and Ca^2+^ Flux in T Cells

**Figure 5:**
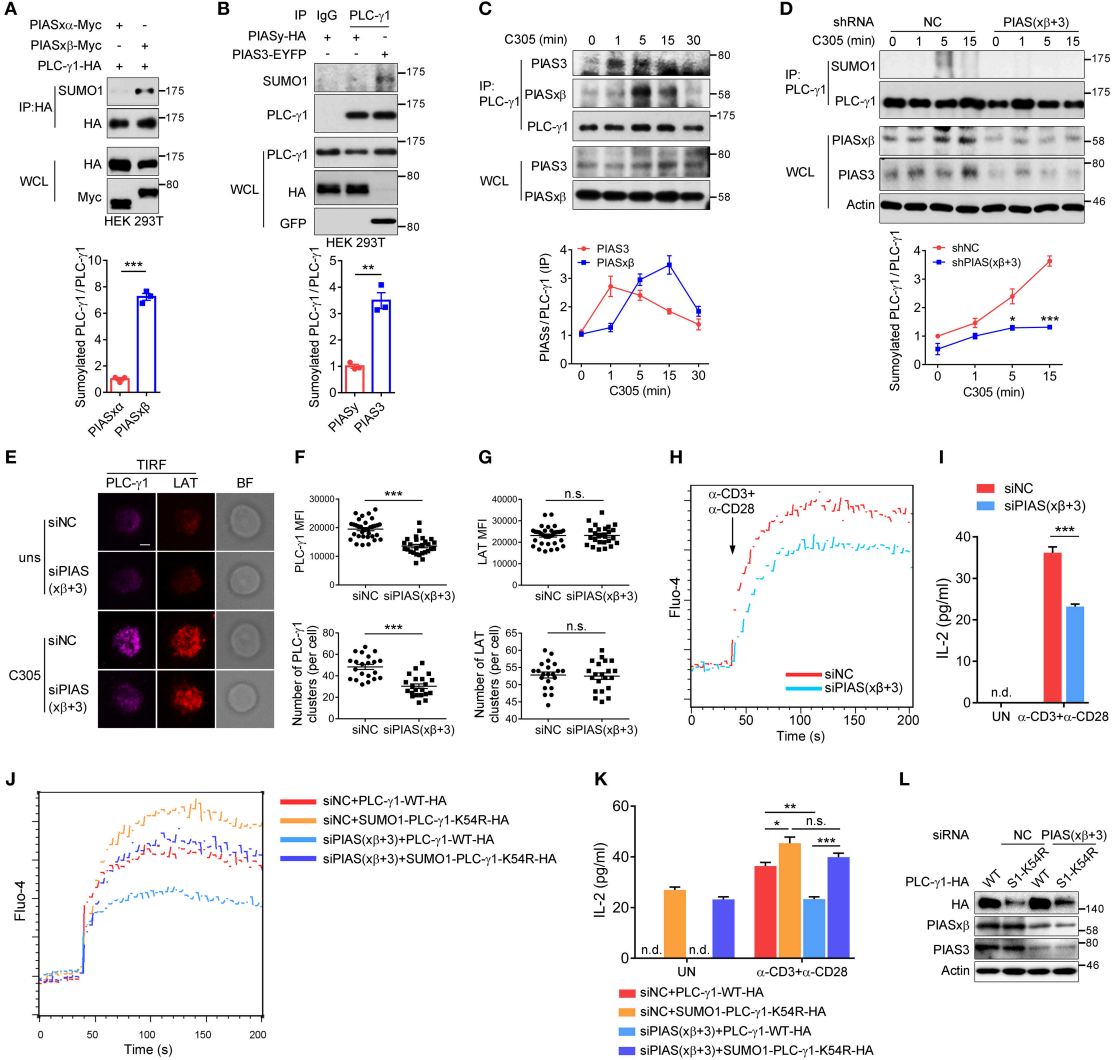
PIASxβ and PIAS3 are the SUMO E3 ligases of PLC-γ1 and regulate PLC-γ1 microcluster formation and Ca^2+^ flux in T cells. **(A,B)** Immunoblot analysis of the sumoylation of PLC-γ1 among proteins immunoprecipitated with anti-HA **(A)** or anti-PLC-γ1 **(B)** from HEK 293T cells transfected with Myc-tagged PIASxα or PIASxβ together with HA-tagged PLC-γ1 **(A)** or transfected with HA-tagged PIASy or EYFP-tagged PIAS3 **(B)**. The densitometric quantification of the ratio of sumoylated PLC-γ1 to immunoprecipitated PLC-γ1 (the ratios for the PIASxα-transfected sample **(A)** and the PIASy-transfected sample **(B)** were set as 1) is shown below. **(C)** Immunoblot analysis of the association between endogenous PIASxβ, PIAS3, and PLC-γ1 among proteins immunoprecipitated with anti-PLC-γ1 from Jurkat E6.1 cells stimulated for 0–30 min with C305. The densitometric quantification of the ratio of immunoprecipitated PIASxβ and PIAS3 to immunoprecipitated PLC-γ1 (the ratio at 0 min was set as 1) is shown below. **(D)** Immunoblot analysis of the sumoylation of PLC-γ1 in Jurkat E6.1 cells transfected with negative control (shNC) or shPIASxβ (short hairpin RNA targeting PIASxβ) and shPIAS3 (short hairpin RNA targeting PIAS3) interference plasmids and stimulated with C305 for 0–15 min. The densitometric quantification of the ratio of sumoylated PLC-γ1 to immunoprecipitated PLC-γ1 (the ratio for the negative control at 0 min was set as 1) is shown below. **(E)** TIRF microscopy of the proximal membrane localization of PLC-γ1 (magenta) and LAT (red) in E6.1 cells transfected with negative control or siPIASxβ and siPIAS3 and left unstimulated (uns) or stimulated with C305 coated on slides for 5 min. Representative images are shown. Scale bar, 4 μm. **(F,G)** Quantification of the TIRF mean fluorescence intensity (MFI) and the number of PLC-γ1 **(F)** and LAT **(G)** microclusters in cells treated with C305. Each symbol represents an individual cell. **(H)** Flow cytometry analysis of the Ca^2+^ flux (fluorescence intensity of Fluo-4) in Jurkat E6.1 cells transfected with negative control or siPIASxβ and siPIAS3 and then stimulated with anti-CD3 and anti-CD28. **(I)** ELISA of IL-2 production in the culture supernatants of Jurkat E6.1 cells transfected with negative control or siPIASxβ and siPIAS3 and then stimulated with anti-CD3 and anti-CD28 for 48 h. **(J)** Flow cytometry analysis of the Ca^2+^ flux (fluorescence intensity of Fluo-4) in Jurkat E6.1 cells transfected with negative control or siPIASxβ and siPIAS3 together with HA-tagged PLC-γ1-WT or SUMO1-PLC-γ1-K54R and then stimulated with anti-CD3 and anti-CD28. **(K)** ELISA of IL-2 production in the culture supernatants of Jurkat E6.1 cells transfected with negative control or siPIASxβ and siPIAS3 together with HA-tagged PLC-γ1-WT or SUMO1-PLC-γ1-K54R and then stimulated with anti-CD3 and anti-CD28 for 48 h. **(L)** Expression of endogenous PIASxβ and PIAS3 and exogenous PLC-γ1-WT and SUMO1-PLC-γ1-K54R in Jurkat E6.1 cells transfected with the indicated plasmids and siRNAs. S1-K54R: SUMO1-PLC-γ1-K54R; n.d.: not detected; n.s.: not significant; ^*^P < 0.05, ^**^P < 0.01, and ^***^P < 0.001 (two-tailed unpaired Student's *t*-test). The data are presented as the mean (± s.e.m.). The data are representative of at least three independent experiments.

The PIAS proteins, including PIAS1, PIASxα, PIASxβ, PIAS3, and PIASy, form the largest family of mammalian sumoylating E3 ligases (35–37). To determine which SUMO E3 ligase catalyses the sumoylation of PLC-γ1, we coexpressed each PIAS with PLC-γ1 in HEK 293T cells. The results showed that PLC-γ1 associated with PIASxβ, PIAS3, and PIASy (Supplementary Figures 5A–C) but not PIASxα (Supplementary Figure 5A). Moreover, both PIASxβ and PIAS3 could catalyse the sumoylation of PLC-γ1 (Figures 5A,B), with PIASxβ being more efficient than PIAS3 (Supplementary Figure 5D). However, PIASy, PIASxα, and PIAS1 did not result in sumoylation of PLC-γ1 (Figures 5A,B and Supplementary Figure 5E). These results suggested that PIASxβ and PIAS3 may both be E3 ligases for PLC-γ1. Next, we investigated whether TCR stimulation induced PLC-γ1 association with PIASxβ or PIAS3. As expected, both PIASxβ and PIAS3 interacted with PLC-γ1 upon TCR stimulation (Figure 5C). However, after TCR stimulation, PIAS3 quickly associated with PLC-γ1 beginning at 1 min, whereas PIASxβ started to associate beginning at 5 min (Figure 5C), indicating that the two proteins had different interaction dynamics. Further, knockdown of either PIASxβ or PIAS3 decreased TCR-induced sumoylation of PLC-γ1, but knockdown of PIASxβ was more efficient than knockdown of PIAS3 in blocking TCR-induced sumoylation of PLC-γ1 (Supplementary Figure 5F), consistent with the above result that PIASxβ sumoylated PLC-γ1 more efficiently than PIAS3 (Supplementary Figure 5D). Upon simultaneous knockdown of PIASxβ and PIAS3 in T cells, TCR-induced sumoylation of PLC-γ1 was almost completely abolished (Figure 5D). Moreover, we analyzed the effects of knockdown of both PIASxβ and PIAS3 on TCR-induced formation of PLC-γ1 microclusters, calcium flux, and IL-2 production in Jurkat T cells. Consistent with the results obtained with the K54R mutant, knockdown of both E3 ligases dramatically impaired TCR-induced PLC-γ1, but not LAT, microcluster formation (Figures 5E–G and Supplementary Figure 5G), Ca^2+^ flux (Figure 5H), and IL-2 production (Figure 5I). Additionally, we investigated whether the impaired Ca^2+^ flux and IL-2 production upon knockdown of both E3 ligases could be rescued with SUMO1-PLC-γ1-K54R. As expected, reconstituted SUMO1-PLC-γ1-K54R rescued TCR-induced Ca^2+^ flux and IL-2 production (Figures 5J–L). Taken together, these observations demonstrate that PIASxβ and PIAS3 are the essential SUMO E3 ligases of PLC-γ1 in TCR signaling.

## Discussion

TCR-proximal signal transduction has evolved with diverse regulatory mechanisms in multiple layers to ensure the precision of various cellular responses and immune outcomes. We have previously discovered that the sumoylation system controls the organization of mature immunological synapses and T cell activation by targeting PKC-θ (11). In this study, we demonstrated that PLC-γ1 is sumoylated predominantly at K54 upon TCR stimulation and that PIASxβ and PIAS3 are the important SUMO E3 ligases for PLC-γ1. Desumoylation of PLC-γ1 inhibited T cell activation by blocking Ca^2+^ Flux via inhibition of the microcluster formation of PLC-γ1 and the interaction of PLC-γ1 with the adaptor proteins SLP76 and Gads. Thus, our investigation revealed a novel mechanism of PLC-γ1 activation, and our results imply that sumoylation controls the assembly of PLC-γ1 membrane microclusters in TCR-proximal signaling.

By respectively, mutating the two conventional sumoylation sites K54 and K987 in PLC-γ1 or fusing SUMO1 to the N- and C-terminus PLC-γ1-K54R mutant, we found that SUMO1 modification on the complete PH domain of PLC-γ1 is important for the microcluster formation and the function of PLC-γ1 in T cells. The complete PH domain in the N terminal of PLC-γ1 is mainly responsible for the interaction with different types of PIPs and PLC-γ1 membrane localization primarily through non-specific electrostatic interactions via a highly positively charged loop (38–40). Interestingly, a prominent NMR structural feature of SUMO-1 is that it displays a positively charged surface on one side and a distinct negatively charged surface on the opposite side (41). Therefore, sumoylation of PLC-γ1 on K54 in PH domain may create a positively charged surface, largely increasing the total positive charge of the PH domain to promote its binding with PIPs-containing membranes. A similar regulatory mechanism has also been reported for PTEN, in which desumoylation of PTEN significantly blocks its association with the phospholipid membrane by electrostatic interaction (42). Different from K54, K987 locates in the TIM barrel (first recognized in triosephosphate isomerase) that is the catalytic domain of PLC-γ1, K987R mutant could slightly decrease the sumoylation and function of PLC-γ1. Nevertheless, K54R mutant did slightly restore the activation defect of PLC-γ1 depletion in T cells, maybe because of the contribution from K987 sumoylation. Thus, although K987 is not the major sumoylation site for the activation of PLC-γ1 upon TCR stimulation, its sumoylation may facilitate the optimal activation of PLC-γ1 probably via modifying the TIM barrel. Intriguingly, when the SUMO1 was fused to the C-terminus of PLC-γ1-K54R mutant, it could not reverse the defects of microcluster formation and Ca^2+^ flux caused by K54R mutation after TCR stimulation. We speculated the fused SUMO1 at C-terminus may be buried in spatial folding of PLC-γ1 or could not promote PLC-γ1 activation because it is not near PH domain. Together, TCR-induced SUMO1 modification of PLC-γ1 at the right placement is important for the activation and function of PLC-γ1 in T cells.

LAT, Gads and SLP76 are required for TCR-mediated activation of PLC-γ1 through recruitment of PLC-γ1 into the LAT signalosome and into the proximity of the cell membrane and through coupling of PLC-γ1 to Itk (5, 7, 43). Interestingly, we discovered that sumoylation of PLC-γ1 was only required for the TCR-induced association of PLC-γ1 with Gads and SLP76, not for its association with LAT. Given that both PLC-γ1 and SLP76 membrane recruitment are dependent on phosphorylated LAT (5, 43, 44), our observation could be explained by the existence of separate LAT-PLC-γ1 and LAT-Gads-SLP76 complexes. The intact tyrosine phosphorylation of LAT and SLP76 after TCR stimulation in PLC-γ1-desumoylated T cells also suggests that LAT-Gads-SLP76 signaling remains intact when PLC-γ1 is desumoylated. However, this explanation introduces a question: if the complexes are separate, whereas desumoylation of PLC-γ1 did not affect the tyrosine phosphorylation on PLC-γ1 Y783 by ItK (27), how does the Itk recruited by LAT-Gads-SLP76 interact with PLC-γ1? In fact, disrupting the protein-protein association between SLP76 and PLC-γ1 does not affect TCR-induced phosphorylation or the function of PLC-γ1 (45). A new mechanism that the activated Zap70 is released from TCRs to disperse TCR signaling has recently been reported (46). Thus, once Itk is recruited to the PIP3-rich membrane, it may either be spatially close enough or be released from SLP76 to phosphorylate PLC-γ1 recruited by LAT (regardless of whether sumoylation has occurred), even though the phosphorylation of desumoylated PLC-γ1, as we observed, may not be optimal. However, as we demonstrated that desumoylation significantly decreased TCR-induced PLC-γ1 microcluster formation (cell membrane localization), it may be difficult for desumoylated PLC-γ1 to interact with membrane Itk (activated). This question could be answered by the discovery that a substantial fraction of LAT present in subsynaptic vesicles is colocalized vertically with microclusters of SLP-76 in immunological synapses (13). Thus, desumoylated PLC-γ1 may colocalize with LAT in subsynaptic vesicles and encounter Itk in or close to SLP-76 microclusters; the direct association of PLC-γ1 with Gads and SLP76 should be more important for PLC-γ1 microcluster assembly than for PLC-γ1 Y783 phosphorylation.

Intensive investigations of microclusters associated with TCR and TCR-proximal signaling protein complexes have revealed that these microclusters are heterogeneous and dynamic and that they form larger clusters through coalescent, reversible, multivalent and synergistic interactions among the proteins in the microclusters (5, 13, 14, 47). These characteristics of microclusters may help to achieve high levels of specificity and sensitivity in TCR signaling. A recent work demonstrated that the assembly of LAT microclusters does not just involve the gain or loss of component molecules; rather, importantly, a protein phase separation occurs when signaling molecules cluster through synthetic multivalent interactions, thus creating a distinct physical and biochemical compartment that facilitates signaling (9). SUMO1 has one positively charged side with five residues of Lys and a negatively charged opposite side with four residues of Glu or Asp together with a negatively charged pocket (nine Glu and one Asp) (41). In addition to sumoylation of PLC-γ1, which may promote the binding of PLC-γ1 to the PIP2-rich membrane via its positively charged side, multivalent ionic bonding between sumoylated PLC-γ1 molecules may also facilitate PLC-γ1 clustering. The result that desumoylation or forced sumoylation of PLC-γ1 decreased or increased PLC-γ1 microcluster formation may reflect multivalent interactions between SUMO1 molecules. Moreover, the impaired interaction between desumoylated PLC-γ1 and SLP76/Gads suggests sumoylation may also promote the multivalent interaction between the SH3 domain of PLC-γ1 and the P-1 domain within SLP76 proline-rich region (48), and in turn facilitate the assembly of PLC-γ1 microclusters. Thus, our results suggest SUMO1 modification may have a crucial role in the microcluster formation of TCR signaling proteins.

In summary, our findings revealed new crosstalk between the sumoylation system and TCR-induced PLC-γ1 activation, implying an important role for SUMO1 modification in microcluster assembly. Further investigation into the role of sumoylation in the regulation of the immunological functions of PLC-γ1 and of other TCR-proximal signaling proteins could provide profound insights into T cell function and offer potential targets for immunotherapy.

## Author Contributions

YL conceived and supervised this study. YL and J-QL designed the experiments. J-QL and Q-LW performed the experiments. B-NG, J-JX, Y-TY, and XL helped with some of the experiments. YL, Q-LW, and J-QL analyzed the data and wrote the manuscript.

### Conflict of Interest Statement

The authors declare that the research was conducted in the absence of any commercial or financial relationships that could be construed as a potential conflict of interest.
