# Supplementary material for: T Cell Receptor (TCR)-Induced PLC-γ1 Sumoylation via PIASxβ and PIAS3 SUMO E3 Ligases Regulates the Microcluster Assembly and Physiological Function of PLC-γ1
Source: Front Immunol. 2019 Feb 28;10:314. doi: 10.3389/fimmu.2019.00314 (PMC6403162; doi:10.3389/fimmu.2019.00314)
Supplement: Supplementary file 1 [file Image_1.pdf]

Supplementary Material

1 Supplementary Figures

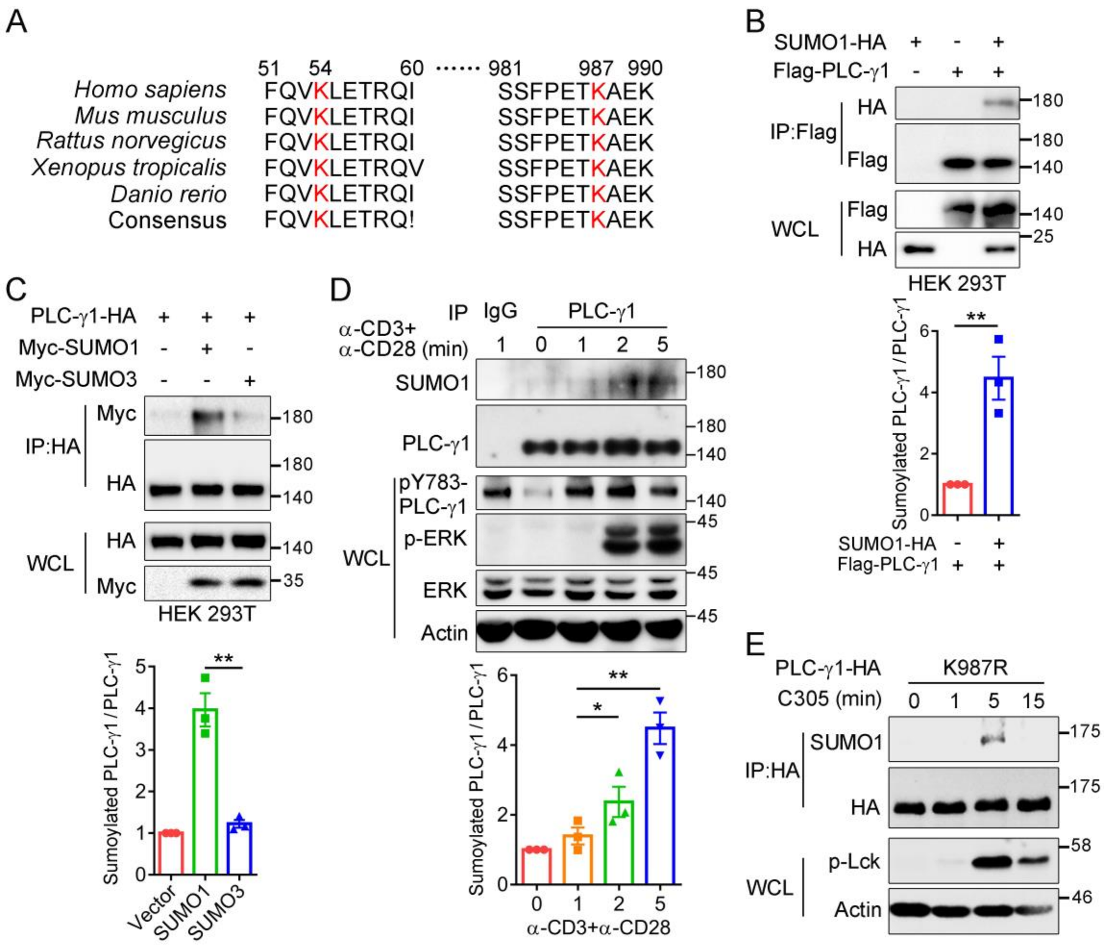

Supplementary Figure 1. PLC-γ1 is modified by sumoylation. (A) Alignment of the sequences near two predicted sumoylation sites (K54 and K987) in some representative species (including *Homo sapiens*, *Mus musculus*, *Rattus norvegicus*, *Xenopus tropicalis*, and *Danio rerio*). **(B)** Immunoblot analysis of the sumoylation of PLC-γ1 in HEK 293T cells transfected with Flag-tagged PLC-γ1 and HA-tagged SUMO1. The densitometric quantification of the ratio of sumoylated PLC-γ1 to immunoprecipitated PLC-γ1 (the ratio for the control was set as 1) is shown below. **(C)** Immunoblot analysis of SUMO modification of PLC-γ1 in HEK 293T cells transfected with HA-tagged PLC-γ1 and Myc-tagged SUMO1 or SUMO3. The densitometric quantification of the ratio of sumoylated PLC-γ1 to immunoprecipitated PLC-γ1 (the ratio for the control was set as 1) is shown below. **(D)** Immunoblot analysis of the sumoylation of PLC-γ1 in Jurkat E6.1 cells stimulated with anti-CD3 and anti-CD28 for 0-5 min. The densitometric quantification of the ratio of sumoylated PLC-γ1 to immunoprecipitated PLC-γ1 (the ratio at 0 min was set as 1) is shown below. **(E)**

Immunoblot analysis of the sumoylation of PLC-γ1 in Jurkat TAg cells transfected with HA-tagged PLC-γ1 K987R mutant and stimulated for 0-15 min with C305. *P < 0.05, and **P < 0.01 (two-tailed unpaired Student's t-test). The data are presented as the mean (± s.e.m.). The data are representative of at least three independent experiments (B-E).

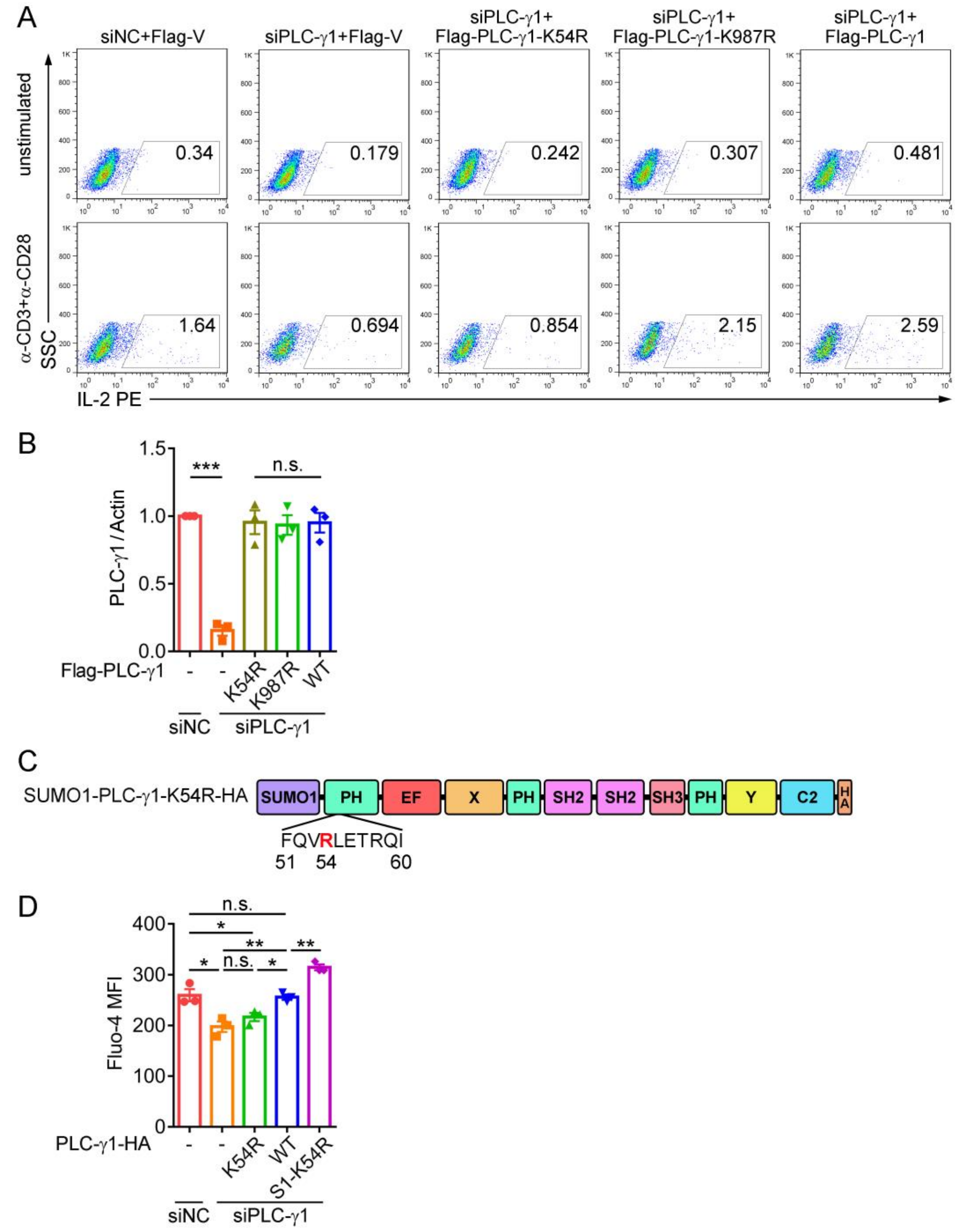

Supplementary Figure 2. PLC-γ1 sumoylation is required for IL-2 production. (A) Representative IL-2 FACS plots of Jurkat TAg cells transfected with Flag-tagged PLC-γ1-WT or KR mutants together with siPLC-γ1 are shown. **(B)** Densitometric quantification of the ratio of PLC-γ1 to actin in Figure 2C (the ratio for the negative control was set as 1). **(C)** Schematic of the structure of SUMO1-PLC-γ1-K54R-HA, a mutant simulating sumoylated PLC-γ1. **(D)** Quantification of the mean fluorescence intensity (MFI) of Fluo-4 in Figure 2D. siNC versus siPLC-γ1, $P = 0.0175$; PLC-γ1-K54R versus siPLC-γ1, $P = 0.2211$. S1-K54R: SUMO1-PLC-γ1-K54R. n.s.: not significant; $*P < 0.05$, $**P < 0.01$, and $***P < 0.001$ (two-tailed unpaired Student's t-test). The data are presented as the mean (± s.e.m.). The data are representative of at least three independent experiments (A, B, and D).

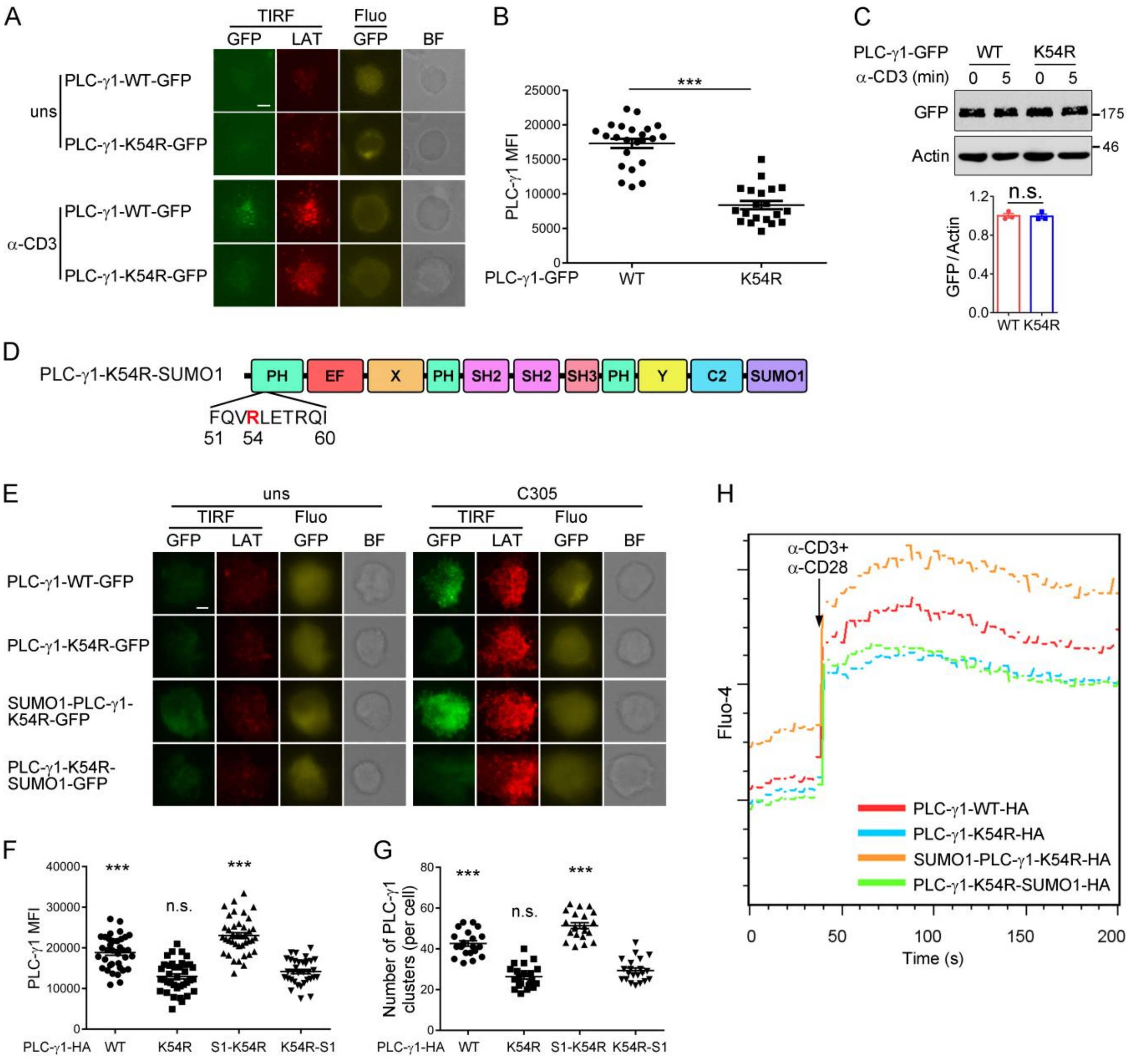

Supplementary Figure 3. Desumoylation of PLC-γ1 diminishes its microcluster formation during T cell activation. **(A)** TIRF microscopy of the proximal membrane localization of PLC-γ1 (TIRF: green, fluorescence: yellow) and LAT (red) in Jurkat E6.1 cells transfected with GFP-tagged PLC-γ1-WT or PLC-γ1-K54R and then left unstimulated (uns) or stimulated with anti-CD3 coated on slides for 5 min. Representative images are shown. Scale bar, 4 μm. **(B)** Quantification of TIRF mean fluorescence intensity (MFI) of PLC-γ1 after TCR stimulation in (A). Each symbol represents an individual cell. **(C)** Expression of GFP-tagged PLC-γ1-WT and PLC-γ1-K54R in (A). The densitometric quantification of the ratio of PLC-γ1-GFP to actin (the ratio of PLC-γ1-WT was set as 1) is shown below. **(D)** Schematic of the structure of PLC-γ1-K54R-SUMO1. **(E)** TIRF microscopy of the proximal membrane localization of PLC-γ1 (TIRF: green, fluorescence: yellow) and LAT (red) in Jurkat E6.1 cells transfected with siPLC-γ1 and HA-tagged PLC-γ1-WT or KR mutants for 48 h and then left unstimulated (uns) or stimulated with C305 coated on slides for 5 min. Representative

images are shown. Scale bar, 4 µm. **(F and G)** Quantification of the TIRF mean fluorescence intensity (MFI) (F) and number of microclusters (G) of PLC-γ1 in cells treated with C305. Each symbol represents an individual cell. **(H)** Flow cytometry analysis of the Ca^{2+} flux (fluorescence intensity of Fluo-4) in Jurkat E6.1 cells transfected with siPLC-γ1 together with HA-tagged PLC-γ1-WT or KR mutants and then stimulated with anti-CD3 and anti-CD28. S1-K54R: SUMO1-PLC-γ1-K54R; K54R-S1: PLC-γ1-K54R-SUMO1. n.s.: not significant; ***P < 0.001 (two-tailed unpaired Student's t-test). The data are presented as the mean (± s.e.m.). The data are representative of at least three independent experiments.

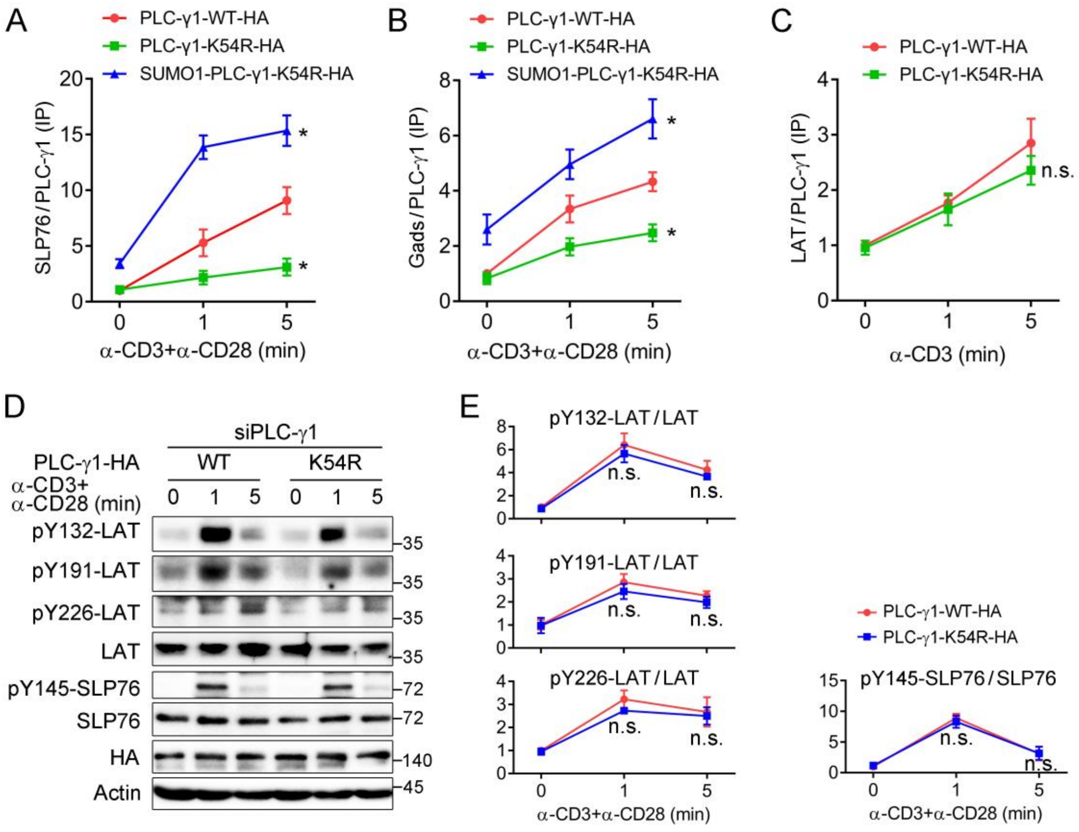

Supplementary Figure 4. Sumoylation of PLC-γ1 promotes its interaction with SLP76 and Gads. (A) Densitometric quantification of the ratio of immunoprecipitated SLP76 to immunoprecipitated PLC-γ1-WT or mutants from Figure 4A and C (the ratio of PLC-γ1-WT at 0 min was set as 1). PLC-γ1-WT versus PLC-γ1-K54R, P=0.0141. **(B)** Densitometric quantification of the ratio of immunoprecipitated Gads to immunoprecipitated PLC-γ1-WT or mutants from Figure 4B and D (the ratio of PLC-γ1-WT at 0 min was set as 1). PLC-γ1-WT versus PLC-γ1-K54R, P=0.0152. **(C)** Densitometric quantification of the ratio of immunoprecipitated LAT to immunoprecipitated PLC-γ1-WT or PLC-γ1-K54R from Figure 4F (the ratio of PLC-γ1-WT at 0 min was set as 1). **(D)** Immunoblot analysis of the phosphorylation of LAT and SLP76 in Jurkat E6.1 cells transfected with siPLC-γ1 and HA-tagged PLC-γ1-WT or PLC-γ1-K54R and then stimulated with anti-CD3 and anti-CD28 for 0-5 min. **(E)** Densitometric quantification of the ratio of phosphorylated LAT to LAT (left) and pY145-SLP76 to SLP76 (right) (the ratio of PLC-γ1-WT at 0 min was set as 1). PLC-γ1-WT versus PLC-γ1-K54R, P = 0.5806 (pY132-LAT, 1min), P = 0.4994 (pY132-LAT, 5min), P = 0.4508 (pY191-LAT, 1min), P = 0.4075 (pY191-LAT, 5min), P = 0.2813 (pY226-LAT, 1min), P = 0.8263 (pY226-LAT, 5min), P = 0.6445 (pY145-SLP76, 1min), and P = 0.9866 (pY145-SLP76, 5min). n.s.: not significant; *P < 0.05 (two-tailed unpaired Student's t-test). The data are presented as the mean (± s.e.m.). The data are representative of at least three independent experiments.

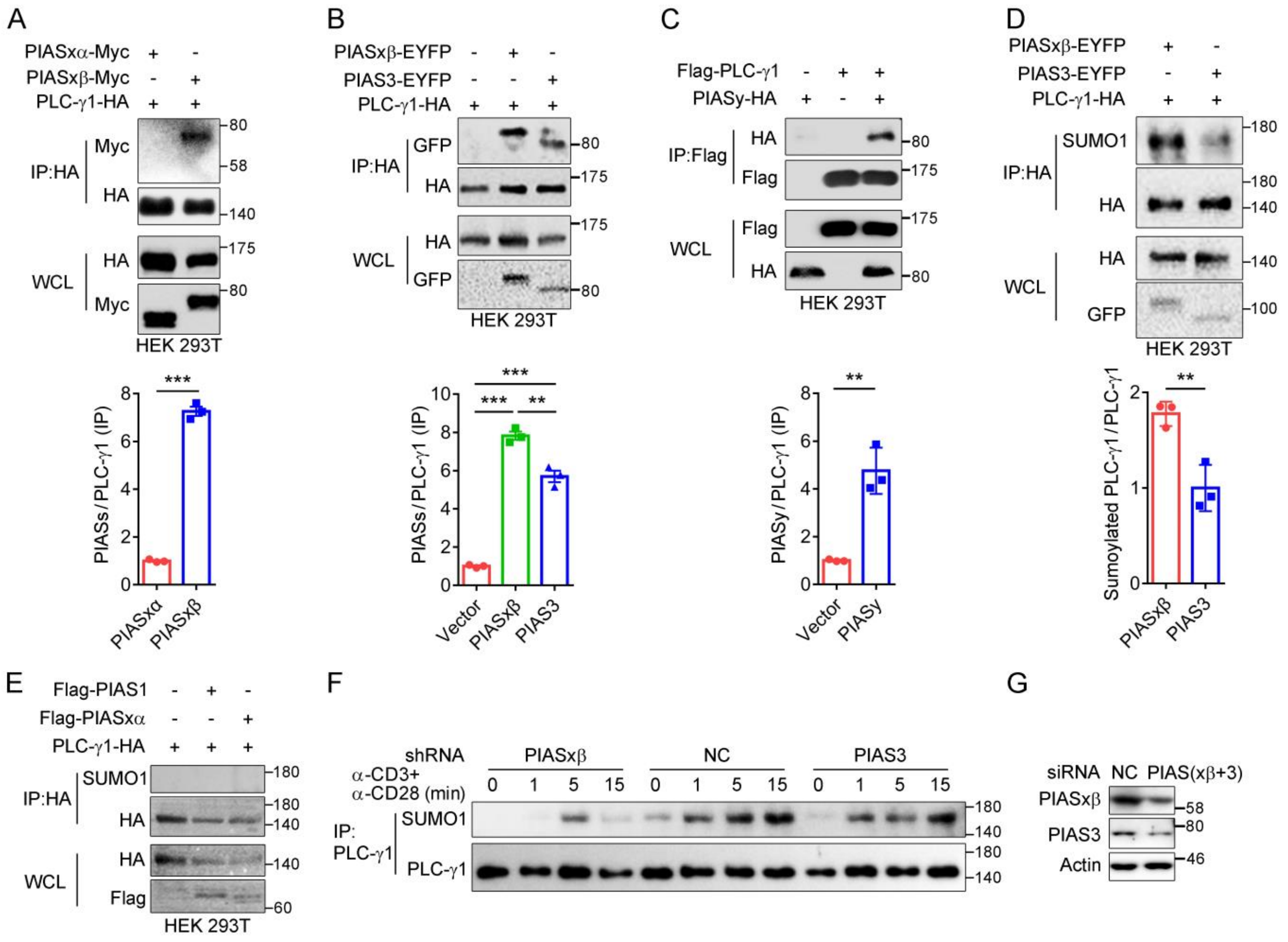

Supplementary Figure 5. PIASxβ and PIAS3 are the SUMO E3 ligases of PLC-γ1. (A-C) Immunoblot analysis of the association of PLC-γ1 with PIAS family members among proteins immunoprecipitated with anti-HA (A and B) or anti-Flag (C) from HEK 293T cells transfected with HA-tagged PLC-γ1 and Myc-tagged PIASxα or PIASxβ (A), transfected with HA-tagged PLC-γ1 and EYFP-tagged PIASxβ or PIAS3 (B), or transfected with Flag-tagged PLC-γ1 and HA-tagged PIASy (C). (A) and Figure 5A are from the same experiment. The densitometric quantification of the ratio of immunoprecipitated PIASs to immunoprecipitated PLC-γ1 (the lowest ratio in each figure was set as 1) is shown at the bottom. **(D and E)** Immunoblot analysis of the sumoylation of PLC-γ1 in HEK 293T cells transfected with HA-tagged PLC-γ1 and EYFP-tagged PIASxβ or PIAS3 (D) and Flag-tagged PIAS1 or PIASxα (E). The densitometric quantification of the ratio of sumoylated PLC-γ1 to immunoprecipitated PLC-γ1 is shown at the bottom (the ratio for the PIAS3-transfected sample was set as 1) (D). **(F)** Immunoblot analysis of the sumoylation of PLC-γ1 in Jurkat E6.1 cells transfected with shPIASxβ or shPIAS3 interference plasmids (with shNC as a negative control) and stimulated with anti-CD3 and anti-CD28 for 0-15 min. **(G)** Expression of endogenous PIASxβ and PIAS3 in E6.1 cells transfected with the indicated siRNAs. $^{**}P < 0.01$ and $^{***}P < 0.001$ (two-tailed unpaired Student's t-test). The data are presented as the mean (± s.e.m.). The data are representative of at least three independent experiments.
